# Supplementary material for: Creating two self-assembly micro-environments to achieve supercrystals with dual structures using polyhedral nanoparticles
Source: Nat Commun. 2018 Jul 17;9:2769. doi: 10.1038/s41467-018-05102-x (PMC6050264; doi:10.1038/s41467-018-05102-x)
Supplement: Supplementary file 1 — Supplementary Information [file 41467_2018_5102_MOESM1_ESM.pdf]

Creating Two Self-assembly Micro-environments to Achieve Supercrystals with Dual Structures using Polyhedral Nanoparticles

Lee et al.

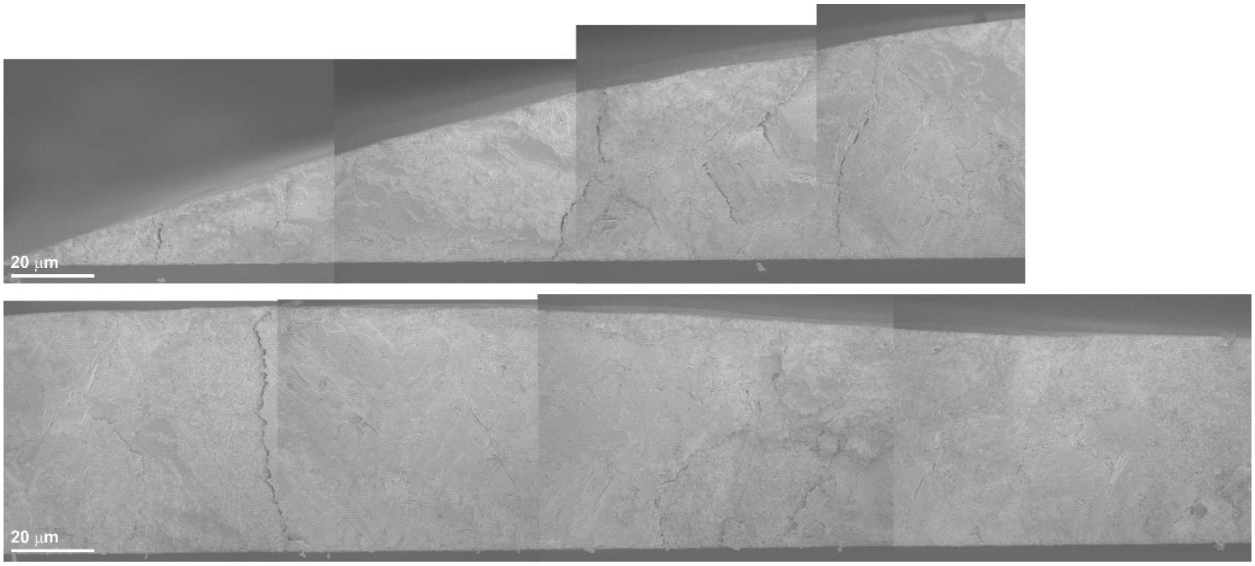

Supplementary Figure 1. Panoramic cross-sectional SEM characterization of the Ag octahedra supercrystals formed using water as the solvent.

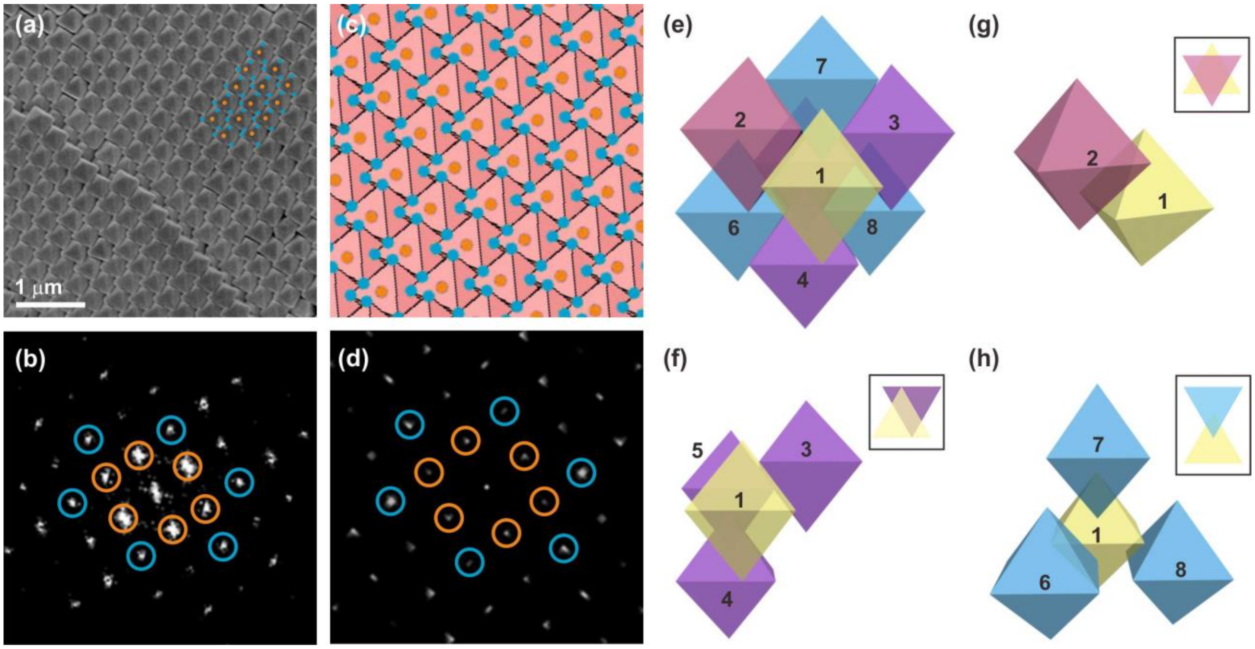

Supplementary Figure 2. Describing the Minkowski lattice. (a-d) The bulk of the supercrystal can be indexed to the Minkowski lattice. Similar octahedra orientation is observed from both the (a) SEM image and (c) ideal Minkowski lattice. The (b) experimental FFT image also match with that of the (c) ideal lattice. (e-h) Illustration of the Minkowski (e) unit cell and the corresponding (g-h) building blocks with different contact areas. The Minkowski lattice is a complex and densest space-filling crystal structure for octahedral particles, with a packing efficiency of 94.7 %. This is a primitive triclinic lattice, with each lattice containing (e) eight neighboring octahedra. Among the eight octahedra, the yellow octahedron labeled 1 in the center is in contact with the remaining seven octahedra. These seven octahedra can be categorized into three different contacting patterns based on the different contact area they share. The largest contact area covering 2/3 of the [111] triangular facets occurs between (g) octahedra 1 and 2 (red), with the facet center in direct contact with each other. The second type of contact occurs between (f) octahedra 1 and 3-5 (purple) with a contacting area of 4/9 of the [111] triangular facets. The tip of the yellow octahedron is in contact with the three purple octahedra at 1/3 of the octahedra edge. (h) The least area of contact with 2/9 of triangle surface arises between the octahedra 1 and 6-8 (blue), with the tips of the yellow octahedron located at the center of the [111] facets of the blue octahedra.

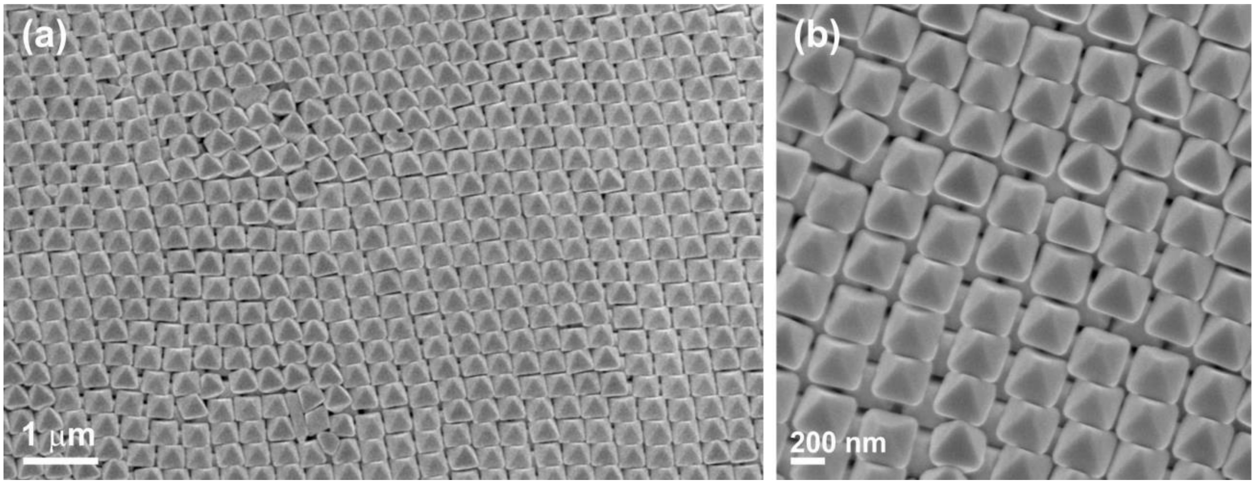

Supplementary Figure 3. Large-area open structures resembling liquid crystalline phase of Ag octahedra on the top layer of the supercrystal. (a) Low- and (b) higher-magnification SEM images.

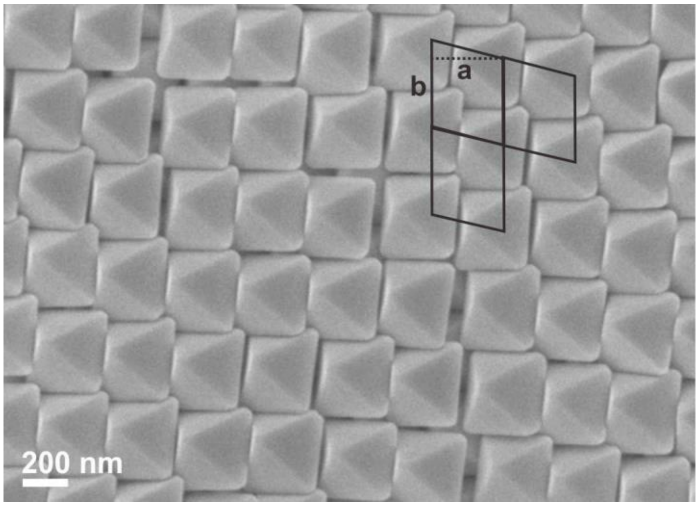

Supplementary Figure 4. Estimating the packing efficiency of the open structure of Ag octahedra. The packing efficiency is estimated to be 49 %. The simplest repeat unit of the open structure corresponds to a rhombic repeat unit as shown above. As with the nature of open structures resembling liquid crystals which lack translational order, the repeat unit changes from area to area. Consequently, the estimated 49 % is likely an overestimate because there are regions with larger voids not taken into consideration.

Supplementary Note 1

Volume of repeat unit = $a \times b \times \frac{\sqrt{6}}{3} x$

$= 377 \times 318 \times 261$

$= 3.129 \times 10^7$ nm^3

a and b are experimentally measured from Figure S4, and x refers to the edge length of the octahedra.

Volume occupied by octahedra in the repeat unit $= \frac{\sqrt{2}}{3} x^3$

$= 1.545 \times 10^7$ nm^3

Packing efficiency $= \frac{1.545 \times 10^7}{3.129 \times 10^7} \times 100$

$= 49$ %

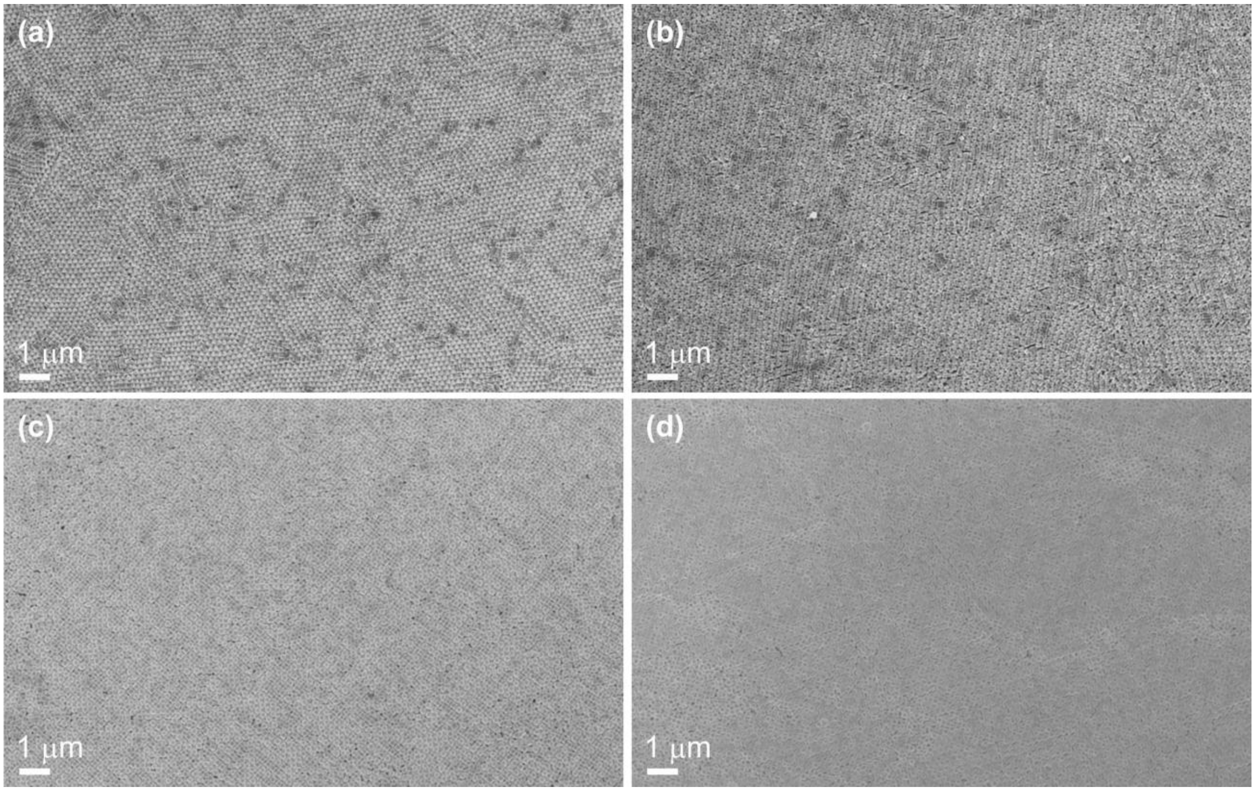

Supplementary Figure 5. Morphology-dependent open structures of Ag polyhedra. Large-area SEM images of the topmost layers for (a) nanocubes, (b) truncated nanocubes, (c) cuboctahedra and (d) truncated octahedra.

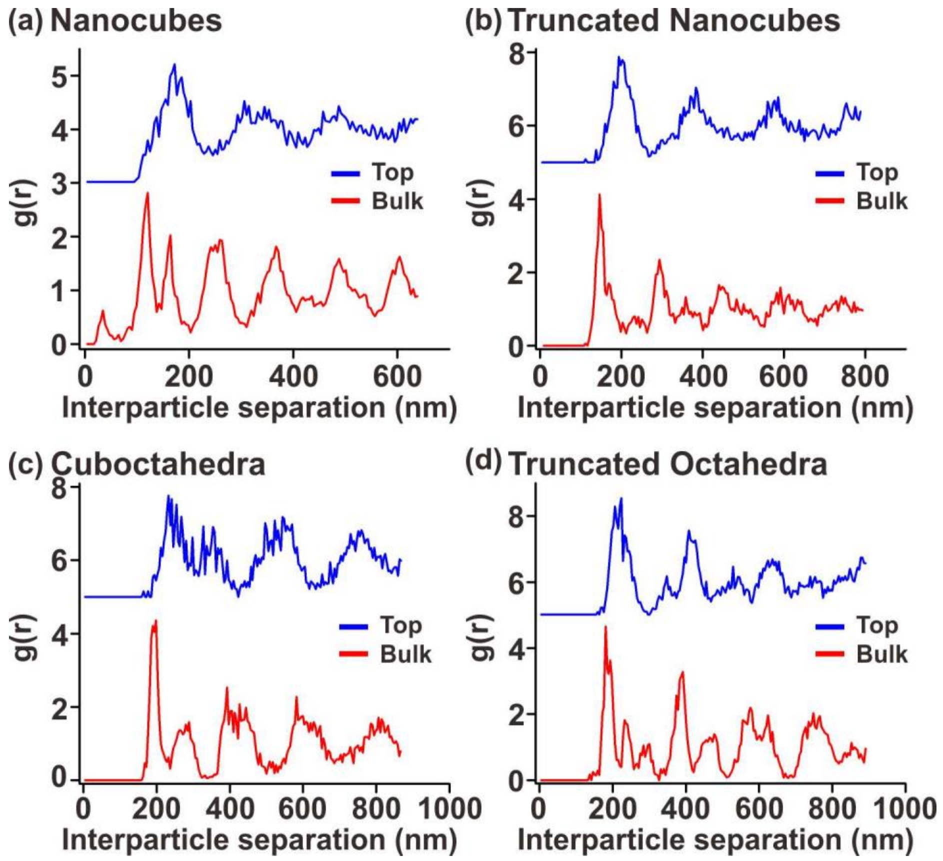

Supplementary Figure 6. Comparing the translational order of open structures and bulk supercrystals. Radial distribution function analyses of (a) nanocubes, (b) truncated nanocubes, (c) cuboctahedra, and (d) truncated octahedra. Blue and red curves correspond to the open structures and bulk supercrystals respectively.

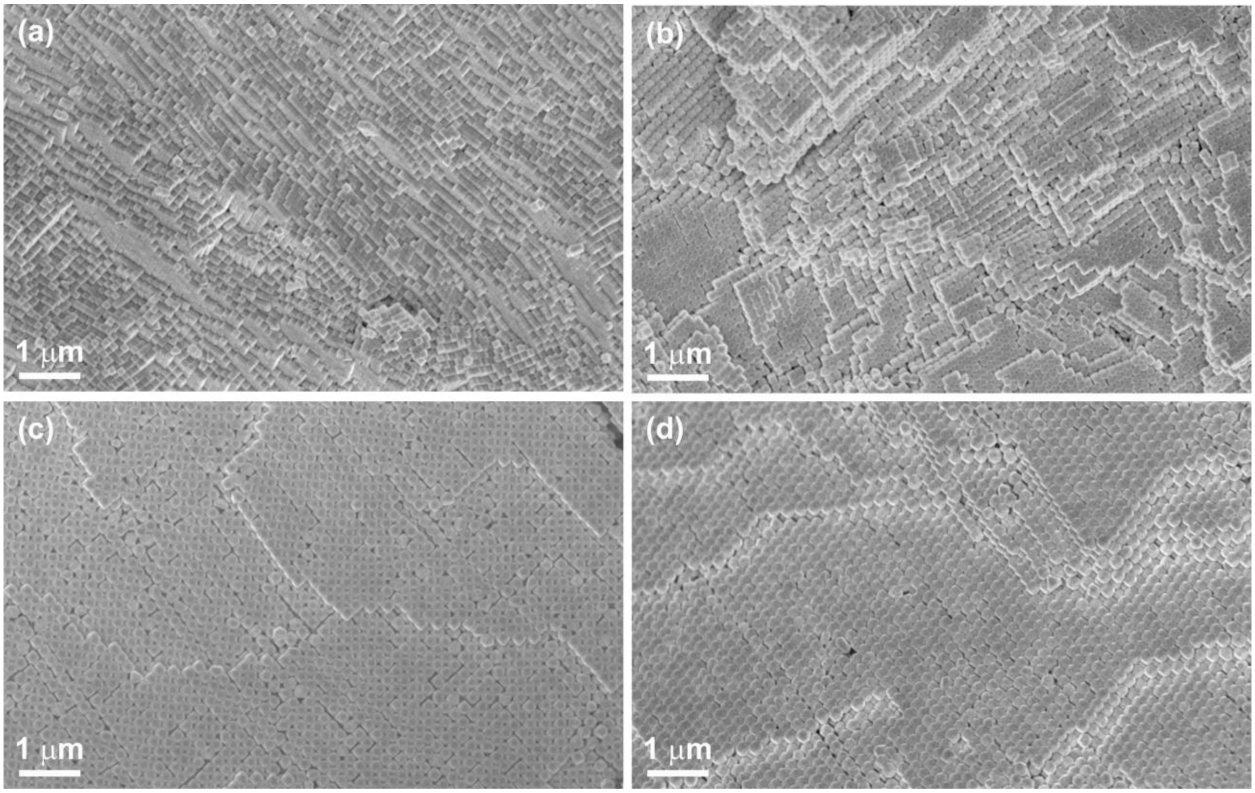

Supplementary Figure 7. Bulk supercrystals of Ag polyhedra. Large-area cross-sectional SEM images of (a) nanocubes, (b) truncated nanocubes, (c) cuboctahedra, and (d) truncated octahedra.

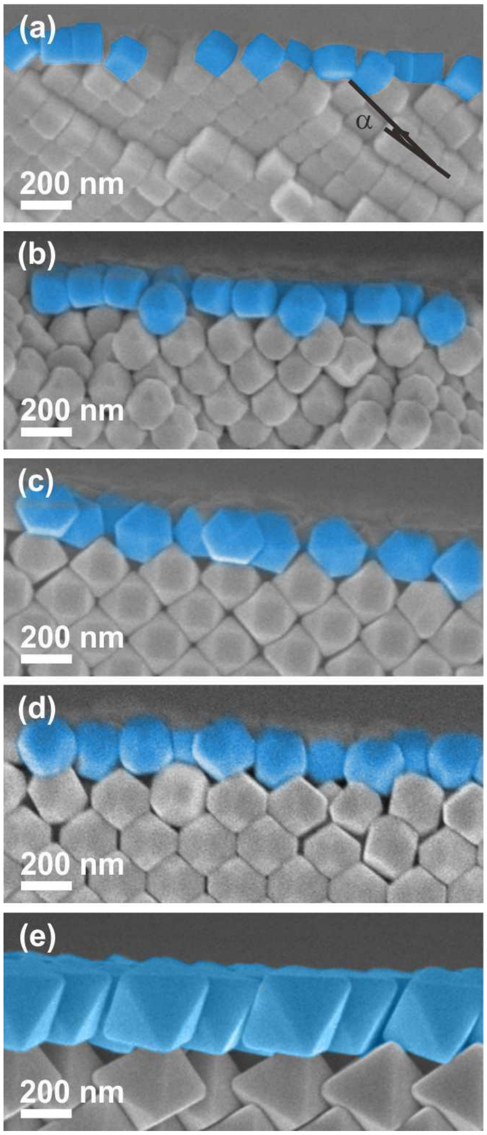

Supplementary Figure 8. Close-up cross-sectional characterization of the break between the topmost layer and bulk supercrystals for various Ag polyhedra. SEM images of (a) nanocubes, (b) truncated nanocubes, (c) cuboctahedra, (d) truncated octahedra, and (e) octahedra indicating the disruption between the topmost layer of nanoparticles (highlighted in blue) and the bulk supercrystal.

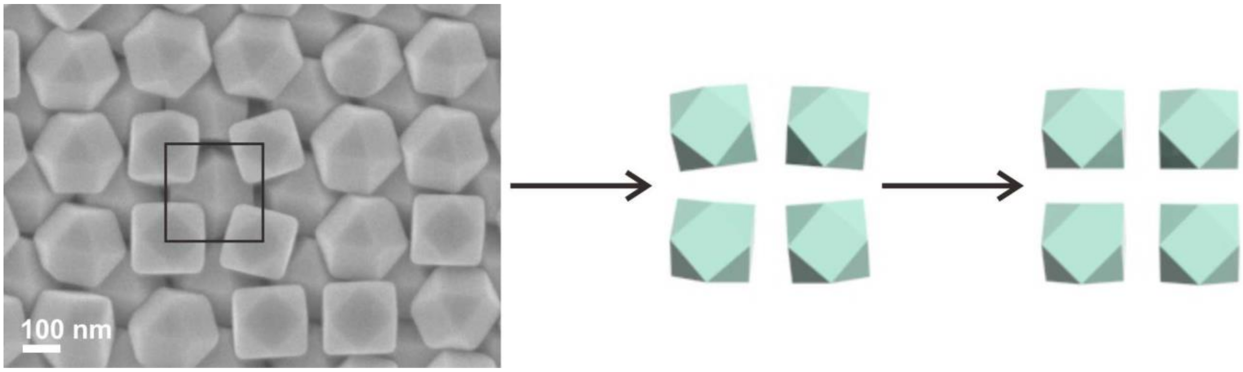

Supplementary Figure 9. Estimating the packing efficiency of the open structure of cuboctahedra. The packing efficiency is estimated to be 42 %. The repeat unit of the open structure corresponds to a square repeat unit as shown above. To simplify the calculations, we assume a more rigid arrangement of the cuboctahedra in a square repeat unit.

Supplementary Note 2

Volume of repeat unit = $2x \times 2x \times \sqrt{2}x$

= $4\sqrt{2}x^3$

Volume occupied by cuboctahedron in the repeat unit = $\frac{5}{3}\sqrt{2}x^3$

x refers to the edge length of the cuboctahedron.

Packing efficiency = $\frac{\frac{5}{3}\sqrt{2}x^3}{4\sqrt{2}x^3} \times 100$

= 42 %

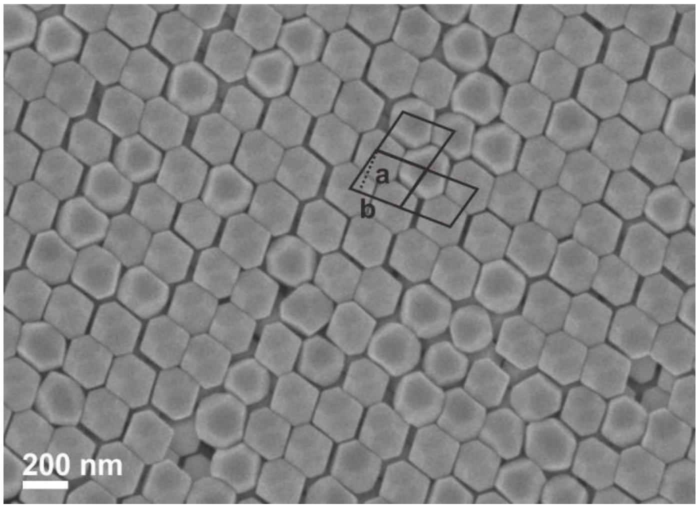

Supplementary Figure 10. Estimating the packing efficiency of the open structure of truncated octahedra. The packing efficiency is estimated to be 46 %. The repeat unit of the open structure corresponds to a rhombic repeat unit as shown above. To simplify the calculations, we assume a more rigid arrangement of the cuboctahedra in a square repeat unit.

Supplementary Note 3

Volume of repeat unit $= a \times b \times (x + 2y)$

$= 224 \times 194 \times 215$

$= 9.343 \times 10^6$ nm^3

Volume occupied by truncated octahedron in the repeat unit

$= 2 \times \left[\frac{1}{3} \times (x + 2y)^2 \times \frac{\sqrt{2}}{2}(x + 2y)\right] - \sqrt{2}y^3$

$= 4.278 \times 10^6$ nm^3

a and b are experimentally measured from Figure S7, x and y refer to the long and short edge lengths of the truncated octahedron.

Packing efficiency $= \frac{4.278 \times 10^6}{9.343 \times 10^6} \times 100$

$= 46$ %

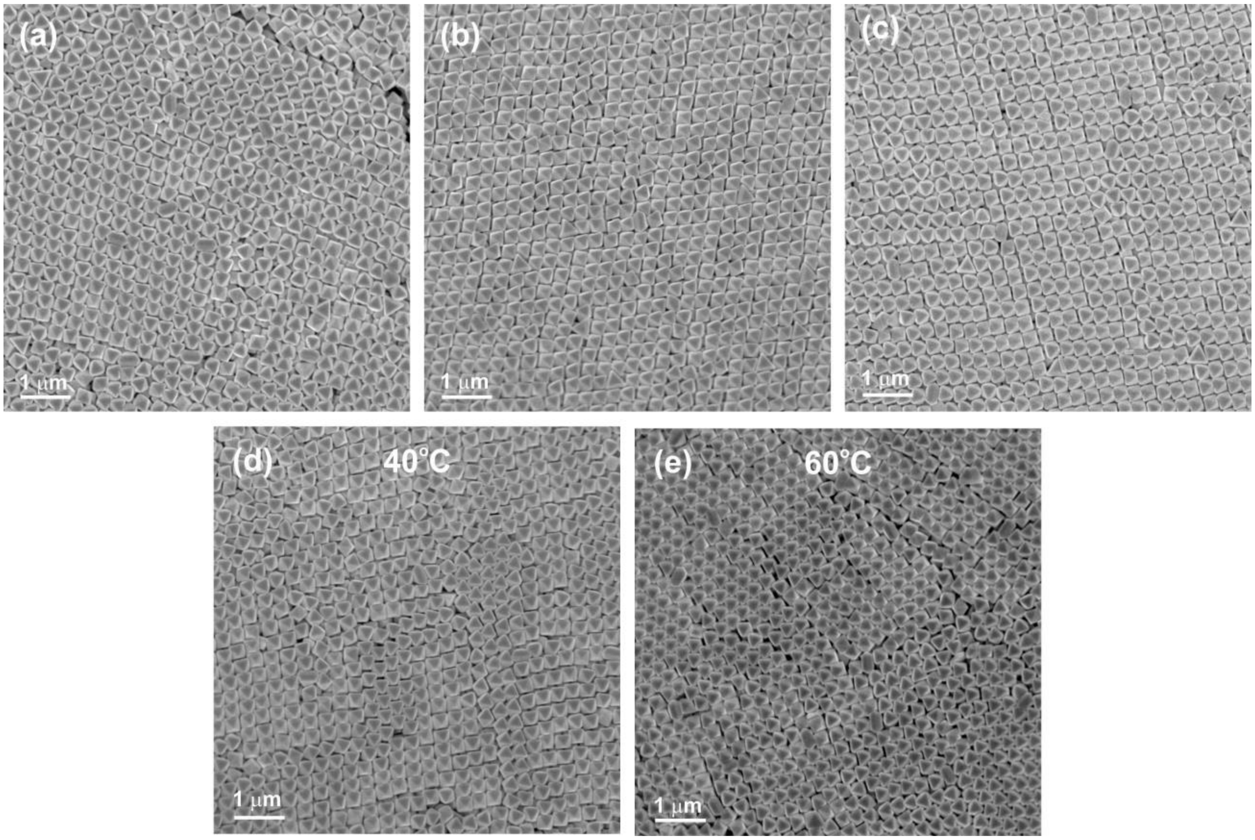

Supplementary Figure 11. Formation of open structures under various experimental conditions for Ag octahedra building blocks. Large-area open structures of Ag octahedra on the top layer of the supercrystal is observed when the (a) droplet is dried upside down, (b) droplet is tilted at an angle, (c) droplet is dried in an environment saturated with water vapor and at elevated temperatures of (d) 40°C and (e) 60°C. In the water vapor-saturated environment, nanoparticle dispersion took ~ 3 days to complete drying. At elevated temperatures, drying is complete within an hour.

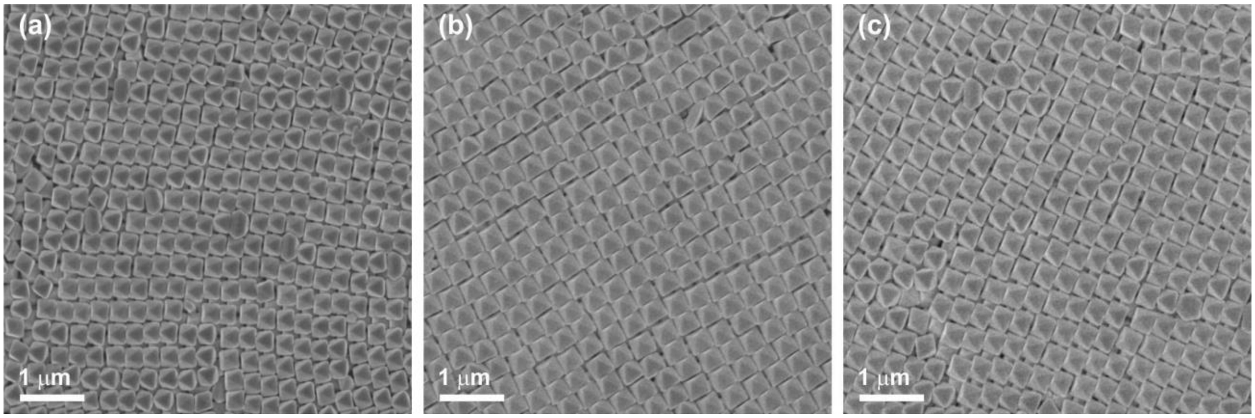

Supplementary Figure 12. Open structure formation is not solvent-sensitive. Formation of open structures in (a) DMF, (b) 1-propanol, and (c) 1-hexanol.

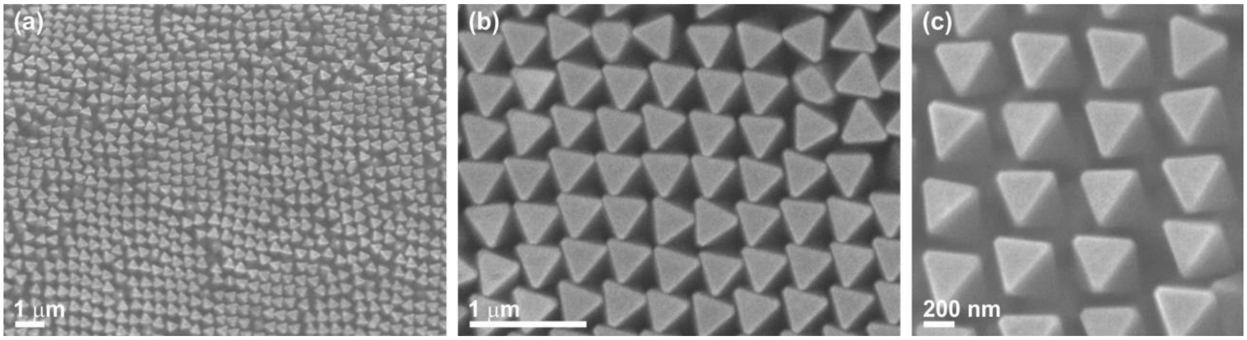

Supplementary Figure 13. Visualizing the organization of Ag octahedra at the air/liquid interface via the polymerization of ethyl cyanoacrylate[1]. The experimental approach involves the formation of a thin polymer film, poly(ethylcyanoacrylate) (PECA), at the air/liquid interface. Ethyl cyanoacrylate monomers are introduced to the droplet of Ag octahedra dispersion via the gas phase in a closed environment. Anionic polymerization of ethyl cyanoacrylate occurs upon contact with the aqueous droplet surface, and is initiated through a nucleophilic attack by water molecules on the ethyl cyanoacrylate monomers. The polymer film grows from the air/liquid interface towards the interior of the water droplet. SEM images at (a) low and (b) higher magnifications. A semi-crystalline monolayer form with emerging translational order during self-assembly. (c) Majority of the Ag octahedra remain embedded within the polymer film to minimize exposure to air.

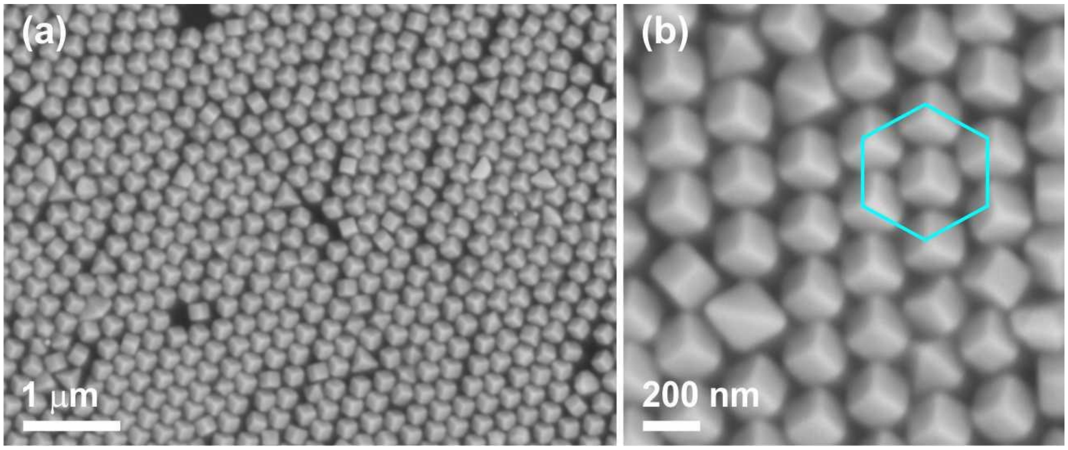

Supplementary Figure 14. Visualizing the organization of Ag nanocubes at the air/liquid interface via the polymerization of ethyl cyanoacrylate. SEM images at (a) low and (b) higher magnifications.

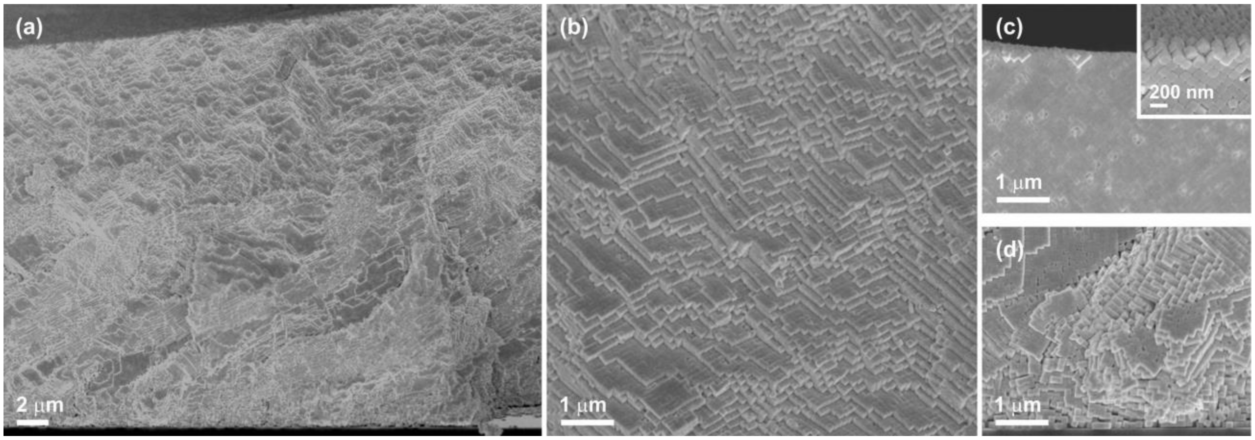

Supplementary Figure 15. Cross-sectional characterization of the assembly of Ag nanocubes. (a, b) Large-area tilt supercrystals are observed. (c) Top- and (d) bottom layers of the assembled supercrystal. The nanoparticle-laden droplet is tilted to one side during drying (similar to the setup in Figure 4b) to create stronger directional convective forces towards the drying front. The SEM images here depict a very 'thick' bulk supercrystal with all the cubes tilted with respect to the substrate normal.

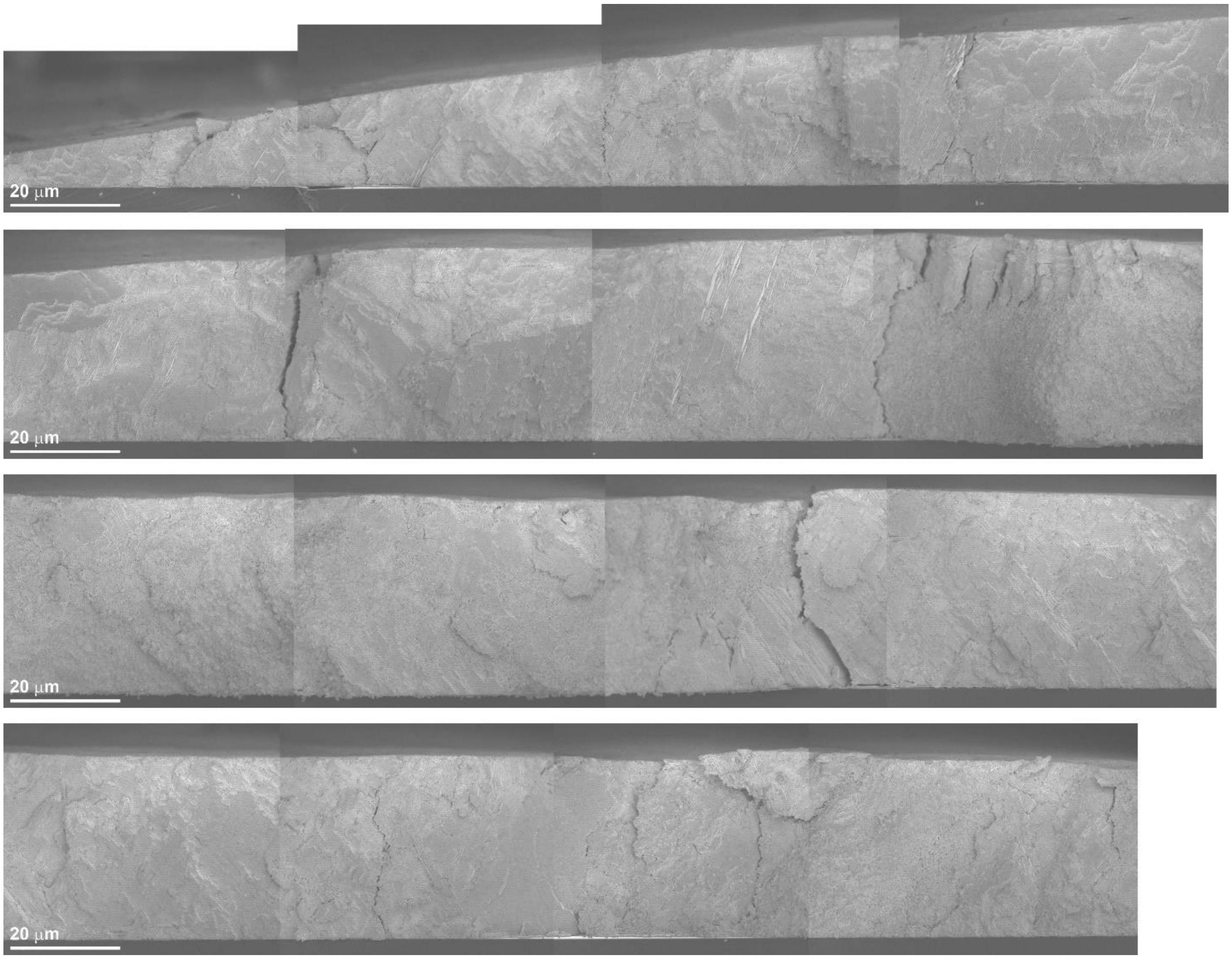

Supplementary Figure 16. Panoramic cross-sectional SEM characterization of the Ag octahedra supercrystals formed using N, N-dimethylformamide (DMF) as the solvent.

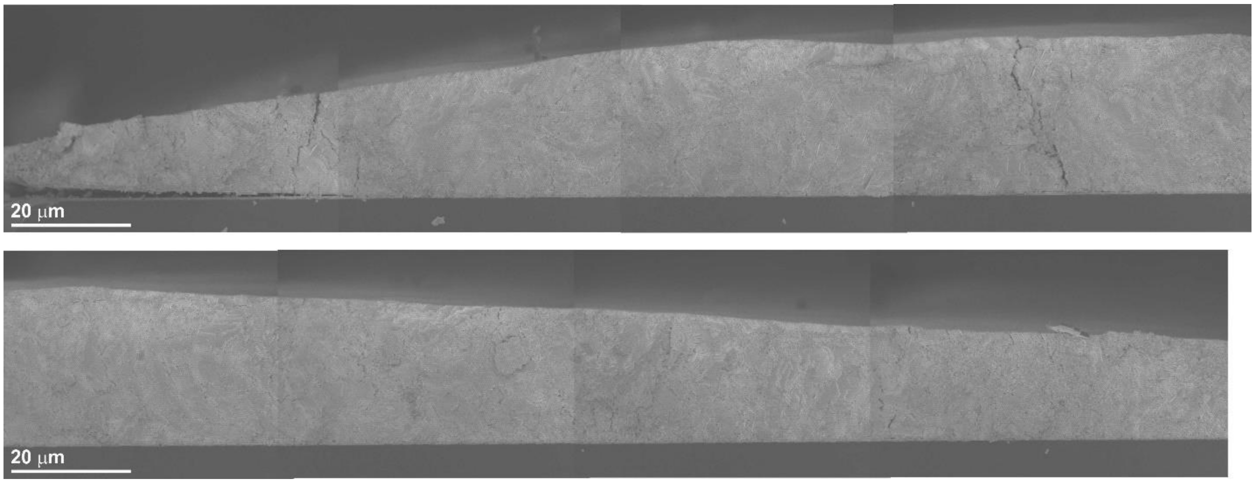

Supplementary Figure 17. Panoramic cross-sectional SEM characterization of the Ag octahedra supercrystals formed using 1-propanol as the solvent.

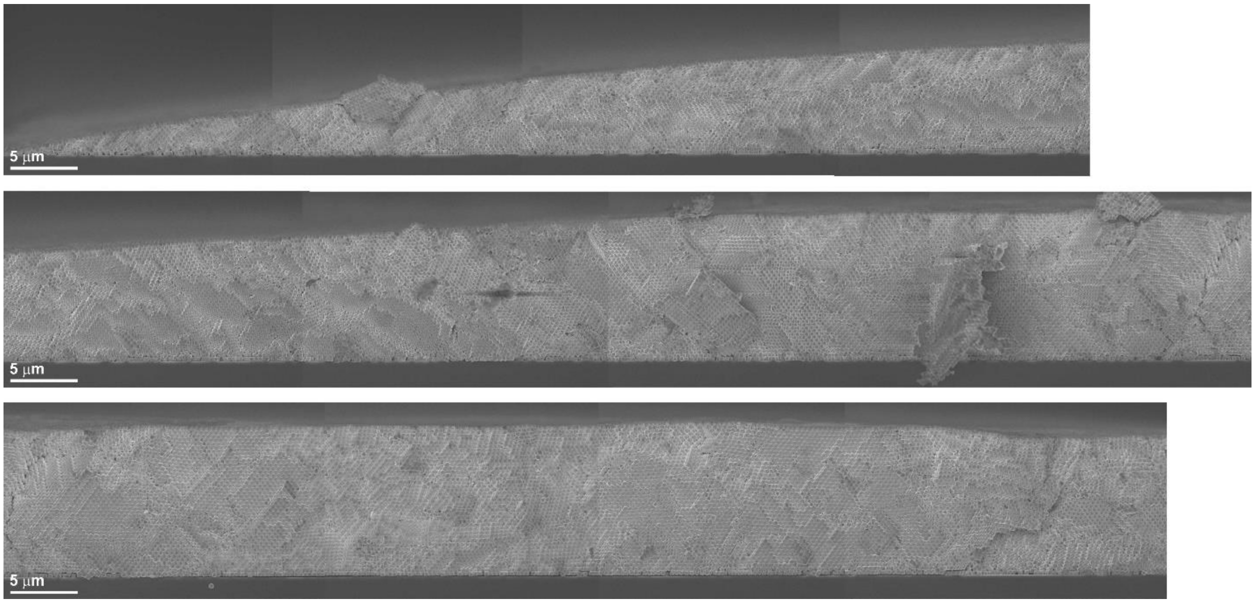

Supplementary Figure 18. Panoramic cross-sectional SEM characterization of the Ag octahedra supercrystals formed using 1-hexanol as the solvent.

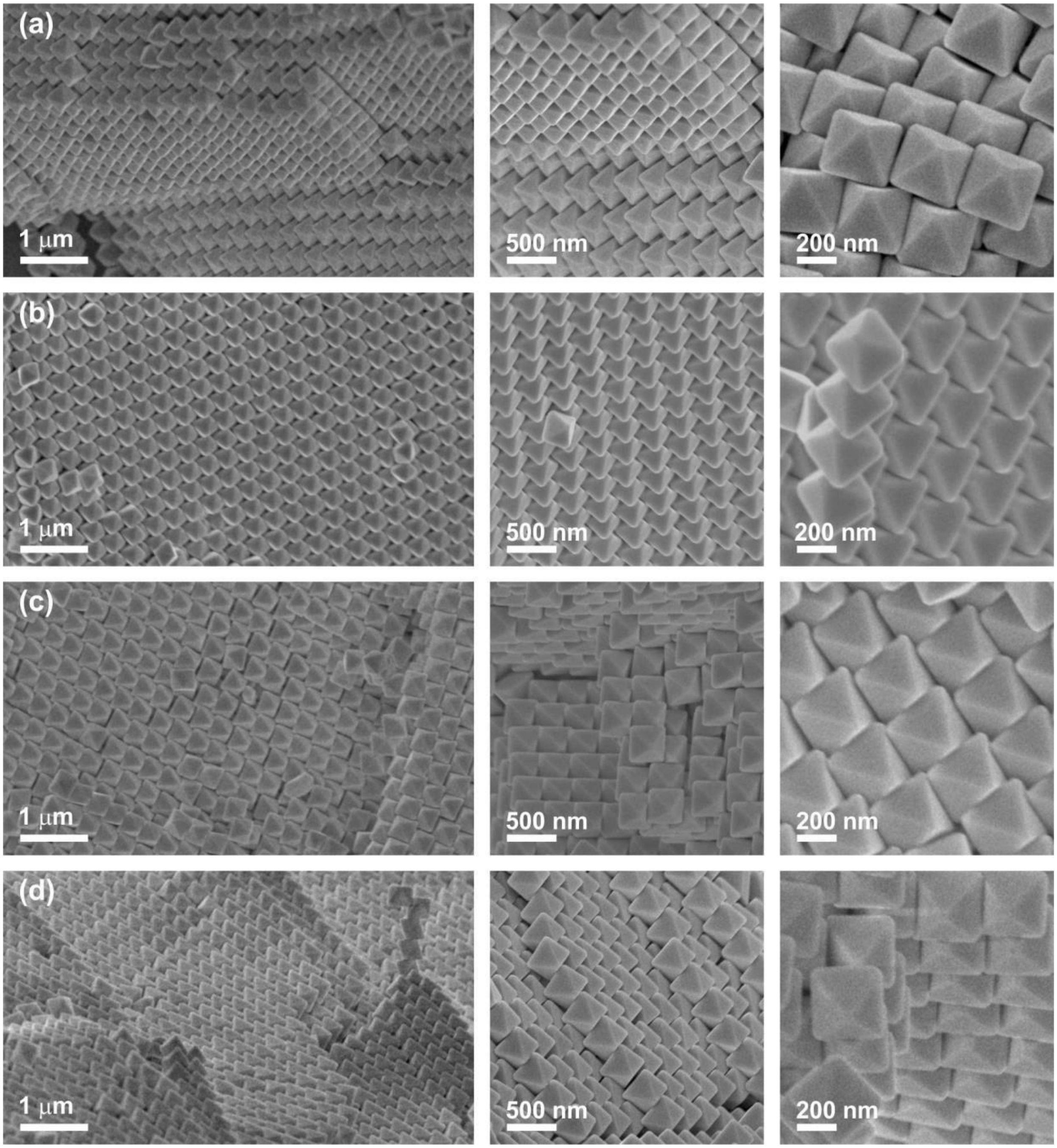

Supplementary Figure 19. Minkowski lattice formation in various solvents. Close-up SEM images of the Minkowski lattice in (a) water, (b) DMF, (c) 1-propanol, and (d) 1-hexanol.

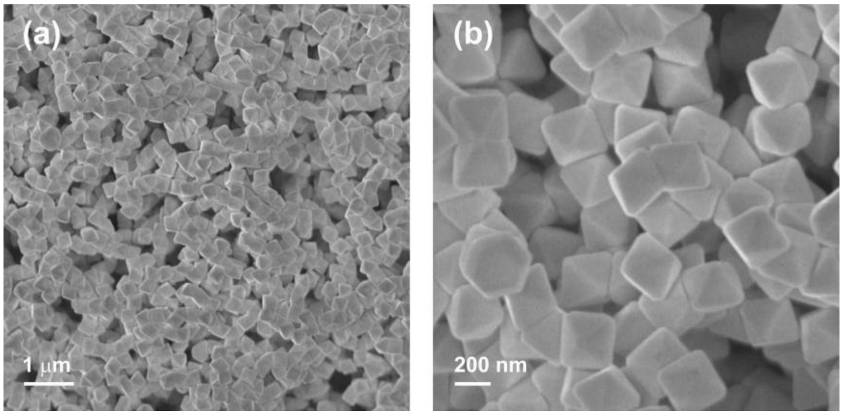

Supplementary Figure 20. PVP-capped Ag octahedra are unable to self-assemble into well-defined supercrystals when toluene is used as the solvent due to the low dispersibility of the Ag octahedra in a non-polar organic solvent. (a) Low- and (b) high-magnification SEM images of the structures formed using Ag octahedra dispersed in toluene.

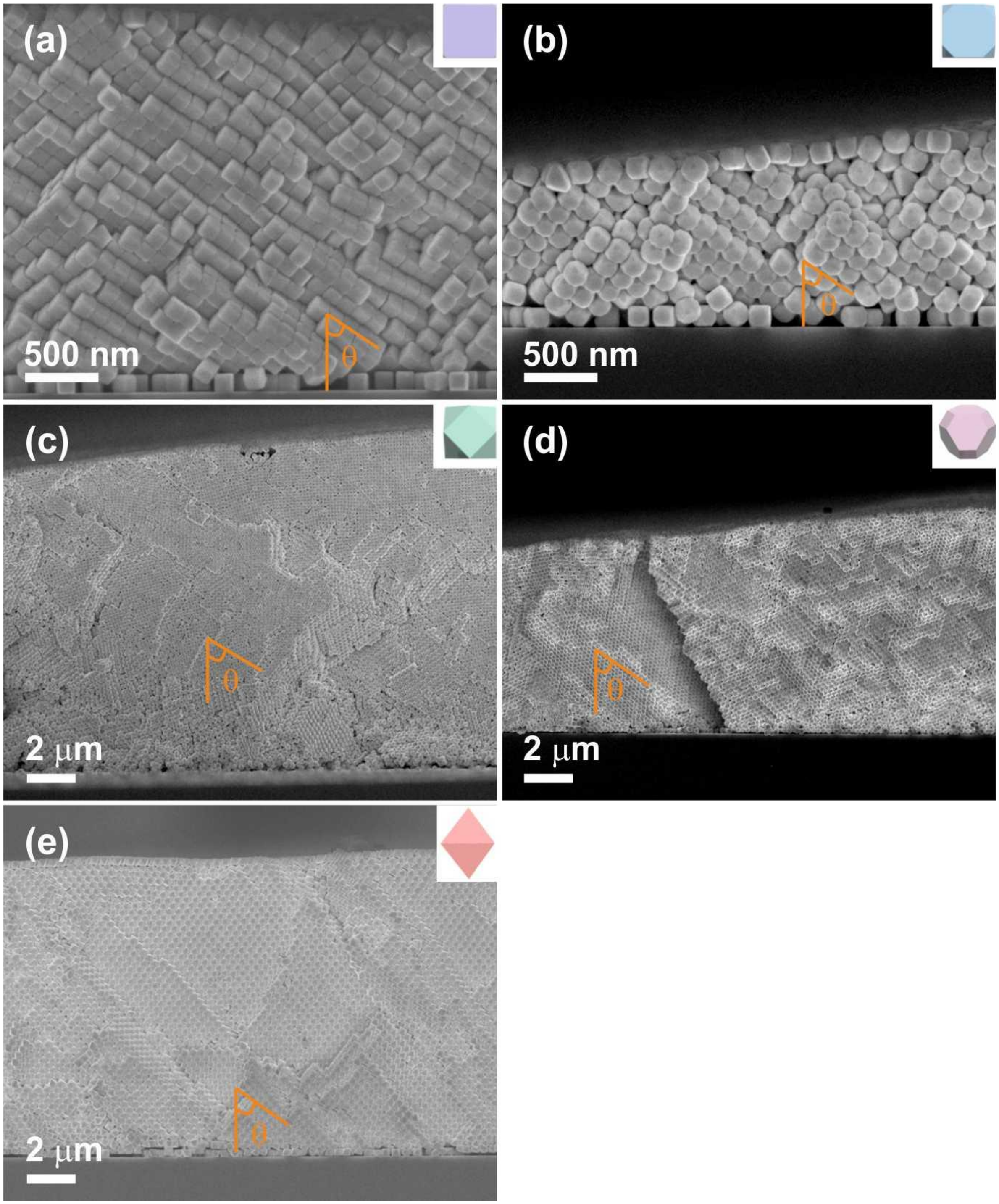

Supplementary Figure 21. Characterizing the supercrystals formed using various shape-controlled Ag polyhedra. A building block-independent tilt is observed from the supercrystals of (a) nanocubes, (b) truncated nanocubes, (c) cuboctahedra, (d) truncated octahedra, and (e) octahedra. Insets are schematics to depict the respective Ag polyhedra. Orange lines illustrate typical tilt angles observed from the supercrystals. The tilt angle ranges from 55 – 60°.

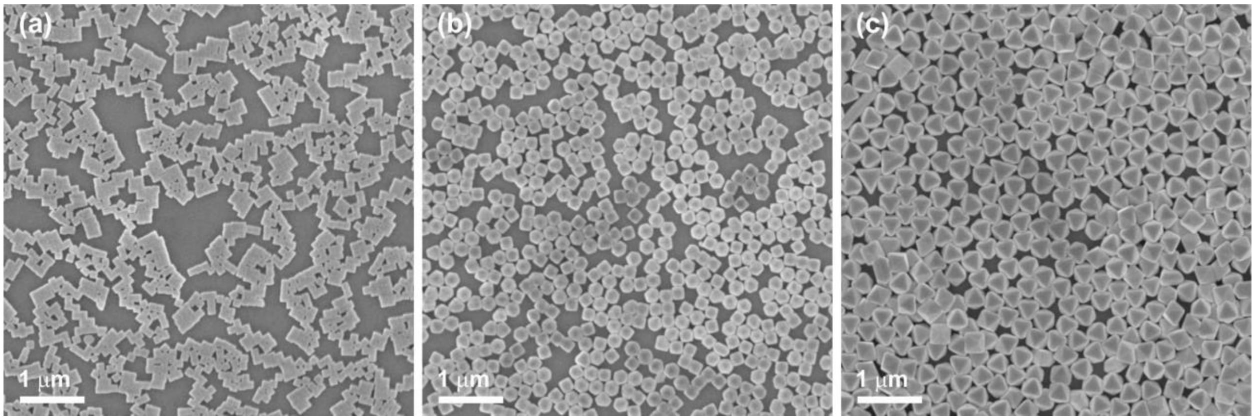

Supplementary Figure 22. Assembled structures using using dilute nanoparticle dispersions. SEM images of (a) nanocubes, (b), cuboctahedra, (c) octahedra for self-assembly.

Supplementary Table 1. Orientational order analysis of the various assembled structures using the respective building blocks.

Morphology	*Cubes*	*Truncated Cubes*	*Cuboctahedra*	*Truncated Octahedra*	*Octahedra*
S_{CD} *(Bulk)*	0.9806	0.8937	0.9820	0.9027	0.9602
S_{CD} *(Topmost layer)*	0.8042	0.8003	0.7446	0.7948	0.9565
% ΔS_{CD}	18	11	24	12	0.4

Supplementary Discussion 1

In plastic crystals, nanoparticles exhibit reduced orientational order while maintaining translational order. On the other hand, nanoparticles in liquid crystals have decreased translational order while maintaining orientational order[2,3]. It should be noted that this decrease in order is relative, and can range from a complete loss in order, to diminished and reduced order.

To analyze the orientational ordering of the metacrystals, we perform additional image analyses using the deuterium order parameter (S_{CD}). S_{CD} is typically used to evaluate the orientational order of hydrocarbon tails in phospholipid bilayers and is calculated using the equation $S = (3\cos^2\theta - 1)/2$. A value of S = 1 indicates perfect nanoparticle orientation along the crystal direction whereas S = 0.5 corresponds to a nanoparticle orientation perpendicular to the crystal direction. S = 0 indicates a random nanoparticle orientation with respect to the crystal direction. θ refers to the angle between the nanoparticle edge vector and the main crystal direction.

To adapt S_{CD} for our nanoparticle-based system, we manually label the nanoparticles in the SEM images to extract the cartesian coordinates. The coordinates are then used to derive one-dimensional edge vectors; these edge vectors represent the orientation of the nanoparticle edge relative to a pre-defined cartesian axis. In our analysis, we choose the main crystal direction of the assembled structure as the default axis. By comparing the edge vectors with the main crystal direction, we can obtain an angle θ to describe the nanoparticle orientation with respect to the main crystal direction. We compare the percentage difference in S values (% ΔS_{CD}) obtained for the topmost layer with that obtained for the bulk supercrystal to correlate the relative loss in orientational order for the topmost layer.

Supplementary Table 2. Change in surface tension in pure and particle-laden solvent droplets. Error bars are s.d. of at least 10 measurements.

Solvent	γ (solvent)	γ (particle)	% decrease
**Water**	72.2 ± 1.3	64.1 ± 0.8	11.2
**N,N-dimethylformamide**	38.7 ± 2.8	33.9 ± 0.7	12.4
**1-Hexanol**	24.3 ± 0.3	22.7 ± 0.1	6.6
**1-Propanol**	23.0 ± 0.1	21.6 ± 0.1	6.1

Supplementary Table 3. Physical properties of the solvents used in the self-assembly of Ag octahedra. Solvent dielectric constants are extracted from the Handbook of Organic Solvents[4].

Solvent	Dielectric Constant
**Water**	79.7
**N,N-dimethylformamide**	36.7
**1-propanol**	20.1
**1-hexanol**	13.3
**Toluene**	2.4

Supplementary Table 4. SERS peak assignment and calculating SERS enhancement factors

SERS Band / cm^{-1}	Vibrational Modes
1080	combination of phenyl ring-breathing, C-H in-plane bending, and C-S stretching
1600	phenyl stretching

Supplementary Note 4

SERS EF = $\frac{I_{SERS}}{N_{SERS}} \div \frac{I_{ref}}{N_{ref}}$

$I_{surface}$ (open structure, octahedra) = 962 counts/s

$I_{surface}$ (supercrystal, octahedra) = 366 counts/s

$I_{solution}$ = 0.109 counts/s (1 M 4-MBT in ethanol)

In solution

$N_{solution}$ = $V_{solution} \times C_{4\text{-MBT}} \times$ Avogadro's number

$= \pi \times \frac{x}{2} \times \frac{y}{2} \times z \times c \times Avogadro's\ number$

$N_{solution}$ = 1.26×10^9 for 1 M 4-MBT in ethanol

Laser resolution in ethanol

x = 910 nm

y = 680 nm

z = 4320 nm

c = 1000 mol/m^3

Laser resolution in air:

x = 520 nm

y = 380 nm

z = 810 nm

Area of laser spot $= \pi \times \frac{x}{2} \times \frac{y}{2}$

$= 1.552 \times 10^{5}\ \text{nm}^2$

$= 1.552 \times 10^{-1}\ \mu\text{m}^2$

Particle density estimated from counting the particle number over areas of more than 50 μm^2 for each assembled structure:

D (open structure, octahedra) = 8.6 octahedra/μm^2

D (supercrystal, octahedra) = 10.6 octahedra/μm^2

Number of Ag polyhedra within the laser spot:

N (open structure, octahedra) = 1.3

N (supercrystal, octahedra) = 1.6

Exposed surface area of Ag polyhedra within the laser spot:

S (open structure, octahedra) $= 4.77 \times 10^{5}\ \text{nm}^2$

S (supercrystal, octahedra) $= 5.88 \times 10^{5}\ \text{nm}^2$

Taking 4.5×10^{14} molecules/cm^2 for a monolayer of 4-MBT on silver [5], number of 4-MBT molecules adsorbed on the particles surface within the laser spot:

N (open structure, octahedra) $= 4.5\ \text{molecules/nm}^2 \times 4.77 \times 10^{5}\ \text{nm}^2$

$= 2.14 \times 10^{6}$

N (supercrystal, octahedra) $= 4.5\ \text{molecules/nm}^2 \times 5.88 \times 10^{5}\ \text{nm}^2$

$= 2.65 \times 10^{6}$

EF (open structure, octahedra) $= \frac{962}{2.14\times10^{6}} \div \frac{0.109}{1.26\times10^{9}}$

$= \underline{5.2 \times 10^{6}}$

EF (supercrystal, octahedra) $= \frac{366}{2.65\times10^{6}} \div \frac{0.109}{1.26\times10^{9}}$

$= \underline{1.6 \times 10^{6}}$

References

1 Vogel, N. *et al.* Direct visualization of the interfacial position of colloidal particles and their assemblies. *Nanoscale* **6**, 6879-6885 (2014).

2 Agarwal, U. & Escobedo, F. A. Mesophase behaviour of polyhedral particles. *Nat. Mater.* **10**, 230-235 (2011).

3 Damasceno, P. F., Engel, M. & Glotzer, S. C. Predictive self-assembly of polyhedra into complex structures. *Science* **337**, 453-457 (2012).

4 Lide, D. R. *Handbook of Organic Solvents*. (CRC Press, 1995).

5 Love, J. C., Estroff, L. A., Kriebel, J. K., Nuzzo, R. G. & Whitesides, G. M. Self-assembled monolayers of thiolates on metals as a form of nanotechnology. *Chem. Rev.* **105**, 1103-1169 (2005).
